# An overview on the nomenclatural and phylogenetic problems of native Asian brine shrimps of the genus *Artemia* Leach, 1819 (Crustacea, Anostraca)

**DOI:** 10.3897/zookeys.902.34593

**Published:** 2020-10-13

**Authors:** Alireza Asem, Amin Eimanifar, Nasrullah Rastegar-Pouyani, Francisco Hontoria, Stephanie De Vos, Gilbert Van Stappen, Shi-Chun Sun

**Affiliations:** 1 Institute of Evolution and Marine Biodiversity, Ocean University of China, 5 Yushan Road, Qingdao 266003, China Ocean University of China Qingdao China; 2 College of Life Sciences and Ecology, Hainan Tropical Ocean University, Yucai Rd, Sanya 572000, China Hainan Tropical Ocean University Sanya China; 3 Independent Senior Research Scientist, Industrial District, 21601 Easton, Maryland, USA Unaffiliated Maryland United States of America; 4 Department of Biology, Faculty of Science, Razi University, 6714967346 Kermanshah, Iran Razi University Kermanshah Iran; 5 Instituto de Acuicultura de Torre de la Sal (IATS-CSIC). 12595 Ribera de Cabanes, Castellón, Spain Instituto de Acuicultura de Torre de la Sal Castellón Spain; 6 Laboratory of Aquaculture & Artemia Reference Center, Faculty of Bioscience Engineering – Blok F, Ghent University, Coupure Links 653, B-9000 Gent, Belgium Ghent University Gent Belgium

**Keywords:** *
Artemia
*, phylogeny, nomenclature, taxonomy, Asia

## Abstract

The genus *Artemia* Leach, 1819 is a cosmopolitan halophilic crustacean, consisting of bisexual species and obligate parthenogenetic populations. Asia is rich in *Artemia* biodiversity. More than 530 *Artemia* sites have been recorded from this area and more than 20 species/subspecies/variety names have been used for them. There exist various problems in the nomenclature, identification, and phylogenetic status of *Artemia* native to Asia, which are discussed in this paper.

The brine shrimp *Artemia* Leach, 1819 is a halophilic zooplankton, distributed in saline habitats worldwide, with the exception of Antarctica (Van Stappen 2002). The genus consists of several bisexual species and a large number of polyphyletic obligate parthenogenetic populations including di-, tri-, tetra-, and pentaploid (Asem et al. 2016). Asia is rich in *Artemia* biodiversity, where more than 530 *Artemia* sites have been recorded (count based on Walter 1888; Sars 1901; Bond 1934; Van Stappen 2002; John et al. 2004; Mura and Nagorskaya 2005; Abatzopoulos et al. 2006; Shadrin and Anufriieva 2012; Salman et al. 2012; Vikas et al. 2012; Zheng and Sun 2013; Naganawa and Mura 2017). Many of the sites are now inhabited by the invasive species *Artemia franciscana* Kellogg, 1906 (in some cases, co-existing with other bisexual species or parthenogenetic populations), whose identification were mostly confirmed by molecular analyses (e.g., Van Stappen et al. 2007; Vikas et al. 2012; Eimanifar et al. 2014). For *Artemia* native to Asia, more than 20 binomial/trinomial names have been used by different authors (see below). Several problems emerged in the past related to the nomenclature, identification or the phylogenetic status of *Artemia* species. Some of these issues have found a solution and scientific consensus, whereas others still persist.

As far as we are aware, 14 binomens (including the one questionably reported as a species of *Branchinecta* Verrill, 1869) and nine trinomens, as well as unidentified species/subspecies/varieties, have appeared in the form of scientific names (combined with a genus name and typeset in italics) in literature relating to native Asian brine shrimps. As shown in Table 1, almost all of the names have some kind of nomenclatural problem. Among the 13 binomens proposed for *Artemia*, eleven fulfil the availability requirements of International Code of Zoological Nomenclature (ICZN, 4^th^ edition) and are considered to be available species names [*Artemia salina* (Linnaeus, 1758); *Artemia asiatica* Walter, 1887; *Artemia urmiana* Günther, 1899; *Artemia parthenogenetica* Bowen & Sterling, 1978; *Artemia sinica* Cai, 1989; *Artemia barkolica* Qian and Wang in Qian et al. 1992; *Artemia urumuqinica* Qian and Wang in Qian et al. 1992; *Artemia ebinurica* Qian and Wang in Qian et al. 1992; *Artemia tibetiana* Abatzopoulos, Zhang & Sorgeloos, 1998; *Artemia frameshifta* Naganawa & Mura, 2017; *Artemia murae* Naganawa in Naganawa and Mura 2017], and the other two are unavailable [*Artemia kazakhastan* Vikas et al., 2012; *Artemia china* Vikas et al., 2012]. *Branchinecta orientalis* Sars, 1901 sensu Chiang, 1983 is supposed to be a misuse for brine shrimp (vide post). Three trinomens (*Artemia sinica sinica* Cai, 1989; *Artemia sinica tibetiana* Abatzopoulos, Zhang & Sorgeloos, 1998; *Artemia salina arietina* Fischer, 1851) are available subspecies names, with all their subspecific names first proposed as (available) specific names. The other six trinomens are unavailable names.

**Table 1.** Names that have ever been used for native brine shrimps *Artemia* of Asia, their availability, information of type specimens and nomenclatural problems

Taxon names	Availability	Type specimens	Type locality	Comments and/or references
*A. salina* (Linnaeus, 1758)	Available	Not mentioned in original description	Salt works at Lymington, England	Linnaeus 1758
*A. asiatica* Walter, 1887	Available	Syntypes. Number of specimens and deposit place not mentioned in original description	Murgab, Tajikistan	This species is known only from type locality (Walter 1887, 1888). It was considered to be a *nomen dubium* (Belk and Brtek 1995)
*A. urmiana* Günther, 1899	Available	Syntypes. Number of specimens and deposit place not mentioned in original description	Urmia Lake, Iran	Günther 1899
*A. parthenogenetica* Bowen & Sterling, 1978	Available	Syntypes, containing cysts from five localities. Deposit place not mentioned in original description	Madras and Kutch, India; Port Hedland, Australia; Sète, France; Yamaguchi-ken, Japan	Though the term “parthenogenetica” was used by earlier authors, Belk and Brtek (1995) clarified its authorship should be Bowen and Sterling (1978). Type specimens should include all specimens that Bowen and Sterling (1978) studied, and type localities include all 5 sites collecting the specimens (ICZN Articles 73.2.3, 76.1). Rogers (2013) listed this name as a nomen dubium.
*A. sinica* Cai, 1989	Available	Syntypes, probably containing cysts and laboratory cultured adults, which are believed to have been lost (Yaneng Cai, pers. comm.)	Yun Cheng Salt Lake, Shanxi, China	*Artemia sinica* was referred to Cai (1989b) in some publications (e.g., Abatzopoulos et al. 1998; Hou et al. 2006; Van Stappen et al. 2009; Zheng and Sun 2013). However, Cai (1989a; an abridged version of Cai 1989b) has nomenclatural priority because it was published earlier (January 1989) than Cai (1989b; spring and fall 1989 / mailed 17 July 1990). Cai (1989a) described only the morphology of adults, but according to Cai (1989b), the type series of this species might contain cysts and laboratory cultured adults (ICZN Article 72.4)
*A. barkolica* Qian & Wang in Qian et al. 1992	Available	“Holotypes” 5♀♀, 5♂♂; paratypes 56♀♀, 4♂♂. Deposited at Xinjiang August First Agricultural College (now Xinjiang Agricultural University), Xinjiang, China	Barkol Lake, Xinjiang, China	Multiple specimens were designated as ‘holotype’ in the original description (Qian et al. 1992). All type specimens may be regarded as syntypes, with original authors’ “holotypes” having the priority in designating as a lectotype when necessary
*A. urumuqinica* Qian & Wang in Qian et al. 1992	Available	“Holotypes” 5♀♀; paratypes 60♀♀. Deposited at Xinjiang Agricultural University	Urumqi Caiwuo Pu Yan Hu (= Dabancheng Salt Lake / Dabancheng Dong Salt Lake), Xinjiang, China	Ibid
*A. ebinurica* Qian & Wang in Qian et al. 1992	Available	“Holotypes” 6♀♀, 2♂♂; paratypes 60♀♀. Deposited at Xinjiang Agricultural University	Ebinur (=Aibi Lake used in many publications), Xinjiang, China	Ibid
*A. tibetiana* Abatzopoulos, Zhang & Sorgeloos, 1998	Available	Syntypes, probably consisting of cysts (two batches collected in different time), nauplii and adults. Deposit place not mentioned in original description	Lagkor Co, Tibet, China	Abatzopoulos et al. (1998) studied adults, nauplii and cysts, all should be components of type series (ICZN Article 72.4). Later studies showed Lagkor Co population was a mixture of bisexual and parthenogenetic *Artemia* (Van Stappen et al. 2003; Maccari et al. 2013). So the type series may contain specimens of more than one species, given that samples studied by Abatzopoulos et al. (1998) were not contaminated after collection and parthenogens were not introduced to the lake after harvesting of type samples
*A. kazakhastan* Vikas et al. 2012	Unavailable	N/A	N/A	This name appeared in Vikas et al. (2012: 135, 138) in the form of binomen, which seemed to refer to “*Artemia* sp. Kazakhstan” mentioned in the same paper. It is obvious that the authors did not intend to establish any new taxa, therefore is unavailable (ICZN Article 16)
*A. china* Vikas et al. 2012	Unavailable	N/A	N/A	Same as the last name, but this name seemed to refer to “*Artemia* sp. China” (Kyêbxang Co population)
*A. frameshifta* Naganawa & Mura, 2017	Available	Holotype ♀; Deposited at Kyoto University Museum	Bajan-Onjul, Tov aimag, Mongolia	Naganawa and Mura 2017
*A. murae* Naganawa in Naganawa & Mura, 2017	Available	Holotype ♂; allotype ♀. Deposited at Kyoto University Museum	Tonkhil nuur (Tonkhil Lake), Gobi-Altai, Mongolia	In addition to the type specimens, Naganawa and Mura (2017) observed 232 other specimens including 124 ♂♂ and 108 ♀♀
? *Branchinecta orientalis* Sars, 1901 sensu Chiang, 1983	N/A	N/A	N/A	Sars (1901) described this anostracan based on specimens from Lake Chuntu-nor, Dornod, Mongolia. Chiang’s (1983) record from Kyêbxang Co, Tibet, China might be *Artemia* (see text)
*A. urmiana parthenogenetica* Barigozzi, 1980 non *A. parthenogenetica* Bowen & Sterling, 1978	Unavailable	N/A	N/A	Barigozzi (1980) used this trinomen as an example to discuss the nomenclature of parthenogenetic *Artemia*. It is unavailable because of no description or diagnosis (ICZN Article 13)
*A. parthenogenetica urmiana* Barigozzi, 1980 non *A. urmiana* Günther, 1899	Unavailable	N/A	N/A	Ibid
*A. sinica sinica* Cai, 1989	See *A. sinica*	See *A. sinica*	See *A. sinica*	Zhou et al. (2003b)
*A. sinica tibetiana* Abatzopoulos, Zhang & Sorgeloos, 1998	See *A. tibetiana*	See *A. tibetiana*	See *A. tibetiana*	Zhou et al. (2003b)
*A. sinica jingyuhuensis* Yin, Zhang & You, 2013	Unavailable	N/A	N/A	Referring to the bisexual population from Jingyu Lake, Xinjiang, China, this name appeared first in the MSc degree thesis of Zhou (2001), and then in Yin et al. (2013). The former was an unpublished work, the later did not describe it as a new taxon and designate name-bearing type(s), thus the name is unavailable (ICZN Articles 13 and 16)
*A. sinica xiaochaidanensis* Yin, Zhang & You, 2013	Unavailable	N/A	N/A	Same as the last name, but referring to the bisexual population from Xiao Qaidam Lake, Qinghai, China
*A. sinica gahaiensis* Yin, Zhang & You, 2013	Unavailable	N/A	N/A	Yin et al. (2013) used this name for the parthenogenetic *Artemia* population from Ga Hai, Qinghai, China. It is unavailable because these authors did not describe it as a new taxon and designate name-bearing type(s) (ICZN Articles 13 and 16)
*A. sinica aibihuensis* Yin, Zhang & You, 2013	Unavailable	N/A	N/A	Same as the last name, but referring to the parthenogenetic *Artemia* population from Ebinur, Xinjiang, China
A. salina var. arietina Fischer, 1851	Available	Syntypes, including several specimens. Deposit place not mentioned in original description	Odessa, Ukraine	Fischer (1851) described *Artemia arietina*, which might be bisexual because both sexes were mentioned. The name is now thought to be a *nomen dubium* (Belk and Brtek 1995). Gurney (1921) reported the Amara (Iraq) population as A. salina var. arietina
*Artemia* sp.	N/A	N/A	N/A	Many Asian *Artemia* populations were reported as *Artemia* sp.
*Artemia s.* subsp. (=*A. sinica* subsp.)	N/A	N/A	N/A	Yin et al. (2011) reported the *Artemia* from Jingyu Lake (Xinjiang, China) and Xiao Qaidam Lake (Qinghai, China) as *A. s.* subsp.
*A. salina* var.	N/A	N/A	N/A	Walter (1888) reported Molla-kary population (Turkmenistan) as “*Artemia salina* L. var.” and considered it a variety between *A. salina* and *Artemia milhausenii* (Fischer, 1834) [“in die Reihe der von *Artemia salina* L. (Milne Edw.) zur *Artemia milhausenii* Fisch.”]. The name *A. milhausenii* was established for *Artemia* from Crimmea (Fischer, 1834) and is now considered a *nomen dubium* or *nomen nodum* (Belk and Brtek 1995; Rogers 2013)

Many Asian *Artemia* populations were reported as *A. salina* in earlier publications but most of these records were later revealed to be parthenogenetic *Artemia*, *A. tibetiana*, *A. sinica* or unidentified bisexual populations (Mura and Nagorskaya 2005; Salman et al. 2012; Shadrin and Anufriieva 2012; Zheng and Sun 2013; Eimanifar et al. 2014; Litvinenko et al. 2016). Even so, the inadequate use of the name still appeared in very recent papers, e.g., Alas et al. (2017) identified the Salt Lake (= Tuz Lake, Turkey) population as *A. salina* although they have been aware that Başbuğ (1999) already documented the population reproducing parthenogenetically.

Padhye and Lazo-Wasem (2018) indicated that the Sambhar Lake (Rajasthan, India) population was a valid report of *A. salina*, whereas the several hundred specimens from this lake at the Indian Museum seem to be all females (Belk and Esparza 1995). Confirmed distribution of this species in Asian countries is restricted to Cyprus, an island in the eastern Mediterranean (Baxevanis et al. 2006).

Bond (1934) reported *A. salina* from Tso Kar, Ladakh, Jammu & Kashmir. Padhye and Lazo-Wasem (2018) studied Bond’s specimens deposited in the Yale Peabody Natural History Museum, and referred them as an *Artemia* sp. that is morphologically close to *A. sinica*. However, this study made a mistake in citing literature and erroneously stated that *A. tibetiana* does not have a basal spine on the male gonopod, even though it is well developed in this species (Mura and Brecciaroli 2004; Zheng and Sun 2008). Given that Tso Kar is closer to the sites of *A. tibetiana* (than those of *A. sinica*), and that they live in similar high altitude habitats, this population awaits a (molecular) comparison with *A. tibetiana*, as well as with other bisexual phylogenetic lineages from adjacent areas such as the “Kazakhstan” and “Kyêbxang Co” population (vide post).

Two varieties of *A. salina* were reported from Asia. Gurney (1921) identified the Amara (Iraq) population as Artemia salina var. arietinaFischer 1851. Since the Amara population is parthenogenetic (Salman et al. 2012), the population is by no means assignable to *A. salina*. Walter (1888) identified his specimens from Molla-kary (Turkmenistan) as “*Artemia salina* L. var.” and thought it to be a variety between *A. salina* and *Artemia milhausenii* (Fischer, 1834). Because Walter’s specimens contained only females, the Molla-kary population may also be parthenogenetic and is not assignable to any bisexual species.

*Artemia urmiana* was originally described as a bisexual species based on specimens from Urmia Lake, Iran (Günther 1899), but Barigozzi and Baratelli (1989) documented that all samples collected from Urmia Lake in 1987 were parthenogenetic and contained di-, tetra-, and pentaploid individuals. Azari Takami (1989) reported that bisexual and parthenogenetic populations coexisted in the Lake. Agh et al. (2007) concluded that parthenogenetic samples had likely been collected from lagoons neighbouring Urmia Lake or its coastal areas, whereas bisexual *A. urmiana* dominated in the main body of the lake. However, a later study documented the existence of parthenogenetic populations in both the lagoons and the main body of Urmia Lake, with significant morphometric differentiation (Asem et al. 2009). In addition to Iran, several populations in Altai (Russia) may belong to this species (Shadrin and Anufriieva 2012), Turkey and Turkmenistan (Eimanifar et al. 2014). The record of this species from Basrah (Iraq) should be *A. franciscana* (see Salman et al. 2012). Outside Asia, Abatzopoulos et al. (2009) identified *A. urmiana* from Lake Koyashskoe (Crimea). Another study based on sequence variation of the mitochondrial COI marker suggested the occurrence of *A. urmiana* in Bulgaria, China, Greece, Crimea, Turkey, and Turkmenistan (Eimanifar et al. 2015). However, these populations need to be further explored with special emphases on the status of reproductive mode (bisexual or parthenogenetic) to confirm the coexistence of *A. urmiana* and parthenogenetic populations in these localities or/and existence of shared COI haplotype(s) between *A. urmiana* and parthenogenetic gene pools.

For two decades, Tibetan bisexual populations have been considered as belonging to a single species, *A. tibetiana*, originally described as a bisexual species from Lagkor Co, Tibet, China (Abatzopoulos et al. 1998). However, as that in Urmia Lake, a parthenogenetic population was also documented from this lake (Van Stappen et al. 2003; Maccari et al. 2013; see Table 1). Wang et al. (2008) documented four Tibetan bisexual populations clustering in two different clades using the mitochondrial COI marker, with one clade only hosting the type locality population (Lagkor Co) and a second distinct clade hosting the others (Kyêbxang Co (=Qixiang Lake or Qi Xiang Cuo), Nima, and Yangnapeng Co). Two other studies have shown that the Tibetan populations clustered in two different groups in a phylogenetic tree based on the COI marker, while all of them represented a single clade based on the nuclear marker ITS1 (Maccari et al. 2013; Eimanifar et al. 2014). Thus, the taxonomic status of these populations awaits to be clarified by future investigations. Chiang (1983) reported *Branchinecta orientalis* from Kyêbxang Co, whereas later studies showed that the anostracan in this lake was *Artemia* (e.g., Zhou et al. 2003a, 2003b; Hou et al. 2006; Yu and Xin 2006; Wang et al. 2008). We suppose that Chiang (1983) might have confused his specimens (no Tb-76-2012) for two reasons: 1) Chiang (1983: 451) reported the altitude of the lake as 4740 m, while it was listed as 4660 m in the chapter “General Account” of the same book (Chiang et al. 1983: 29); and 2) during the time (1976) he collected specimens, the salinity of this lake was as high as 63.27 g/L (Zheng et al. 2002), salinity that is not suitable for *B. orientalis*.

The validity of *Artemia sinica*, described based on specimens from Yuncheng Salt Lake, China (Cai 1989a) has rarely been questioned, and nearly 30 bisexual populations from China (see review of Zheng and Sun 2013), and several populations from Russia (Shadrin and Anufriieva 2012; Litvinenko et al. 2016), and Mongolia (Gajardo and Beardmore 2012; Eimanifar et al. 2014) have been identified as this species so far. The molecular clock divergence analysis indicated that *A. sinica* had already diverged in the late Miocene (19.99 Mya), whereas *A. urmiana*, *A. tibetiana*, and “Eurasian Haplotype Complex” (EHC refer to group of parthenogenetic *Artemia* lineages) shared a common ancestor in the late Pliocene (5.41 Mya) (Eimanifar et al. 2015).

Another controversial topic in taxonomy of *Artemia* relates to a batch of bisexual *Artemia* cysts from Kazakhstan (KAZ; ARC no. 1039), supplied by Catvis Co. (s-Hertogenbosch, The Netherlands) in 1988. However, no information about the exact origin(s) of the sample (Pilla and Beardmore 1994; Ben Cattel, pers. comm. 2017) was provided. An earlier study documented morphological differentiations between this bisexual sample and other Asian species (Pilla and Beardmore 1994). In molecular analyses, this population was located in a separate phylogenetic clade in the mitochondrial COI tree (Maccari et al. 2013), but was clustered together with *A. urmiana* and *A. tibetiana* using ITS1 nuclear marker (Vikas et al. 2012). Under these circumstances, the systematic position and geographical origin of this sample remains a source of debate. So far only one site, the salt lake Margen-sor (district of Atbassar), was reported to be inhabited by bisexual *Artemia* in Kazakhstan (Sars 1901: reported as *A. salina*). Future sampling in Margen-sor may help to elucidate if KAZ and the Margen-sor population belong to the same species/lineage.

Qian et al. (1992) described three species based on specimens from three different salt lakes in Xinjiang, China, namely *Artemia barkolica* Qian & Wang, 1992, *Artemia urumuqinica* Qian & Wang, 1992 and *Artemia ebinurica* Qian & Wang, 1992 (Table 1). Except for 12 papers in the special Chinese-language issue “Studies on *Artemia* of Barkol Lake” (Journal of August 1^st^ Agriculture College, 1994, Vol. 17 no. 2) and Qian et al. (1993), which used the names *A. barkolica* and *A. urumuqinica*, respectively, these three species have not been recognised by other researchers. As commented by Zheng and Sun (2013), the very biased sex ratios in the original description, as well as the results of many other studies, have indicated that all three populations were parthenogenetic. Since all these populations consist of strains of different ploidies (for reference review see Zheng and Sun 2013), and diploid/triploids and tetraploid/pentaploids are assumed to have originated separately (Maniatsi et al. 2011; Asem et al. 2016); each of the three nominal species may represent more than one phylogenetic clade (or species) or they may be synonyms.

Recently, in Mongolia, two new species have been described: *A. frameshifta* and *A. murae* (see Naganawa and Mura 2017). These species have been described using primary morphological characters and a single COI sequence, whereas morphometric differentiation and population genetic analysis have not been studied. Although both species were said to reproduce bisexually in the original descriptions, males have not been observed in *A. frameshifta*. Considering the ‘sex ratio’ (125 males and 109 females were observed), phylogenetic position (sister to *A. sinica*) and genetic distance (p-distance between *A. murae* and *A. sinica* is 4.8%) (Naganawa and Mura 2017), *A. murae* may represent a lineage close to *A. sinica*. Moreover, no sequences of parthenogenetic *Artemia* were included in the phylogenetic analysis of Naganawa and Mura (2017). Therefore, the taxonomic status of these species also needs to be re-confirmed by future multidisciplinary studies on their biology and phylogeny.

*Artemia asiatica* was described according to only female specimens from Murgab, Tadjikistan (Walter 1887). It may be a parthenogenetic population, and the name is now treated as a nomen dubium (Belk and Brtek 1995).

Theoretically, the ability of interfertility and producing offspring able to reproduce is a common criterion to confirm subspecies status (Mayr 1969). In nature however, subspecies populations mostly have an allopatric distribution. Due to geographical isolation, proof of natural interbreeding is practically impossible except in rare cases. The results of cross-breeding tests with different Asian bisexual *Artemia* were inconsistent among different studies. Pilla and Beardmore (1994) documented complete interfertility among *Artemia* sp. (the KAZ sample), *A. urmiana*, and *A. sinica*. Zhou et al. (2003b) showed interfertility between *A. sinica* and *A. tibetiana*, and considered them as subspecies (*Artemia sinica sinica* and *Artemia sinica tibetiana*). In other studies, however, an isolating barrier was found between *A. urmiana* and *A. sinica* (see Zheng and Sun 2008), and between *A. sinica* and *A. tibetiana* (see Abatzopoulos et al. 2002b; Zheng and Sun 2008). Due to the possible effect of laboratory rearing conditions (ionic composition of the medium, salinity and temperature) on the reproductive potential of *Artemia* (Abatzopoulos et al. 2003; Velasco et al. 2016, 2018), it seems that a fertility test is not suitable as a single biological tool to determine *Artemia* population/species ranks. Although reproductive isolation in captivity/laboratory circumstances might refer to a lack of gene flow in nature, laboratory cross-fertility even for F1 and later generations cannot evidence that such cross-fertility would occur under natural conditions if there is no hybrid zone (Helbig et al. 2002).

Two other bisexual populations, the Jingyu Lake (Xinjiang, China) population and the Xiao Qaidam Lake (Qinghai, China) population, were considered to represent different subspecies of *A. sinica*. The subspecies names *Artemia sinica jingyuhuensis* Yin, Zhang & You, 2013 and *Artemia sinica xiaochaidanensis* Yin, Zhang & You, 2013 were proposed for them, respectively, though they are not available (Table 1). Zheng and Sun (2008) documented some morphological differences between Jingyu Lake and Lagkor Co populations though they identified the former population as *A. tibetiana*. In phylogenetic analyses the Jingyu Lake population located in a clade containing *A. tibetiana* (Wang et al. 2008; Yin et al. 2011, 2013), while the Xiao Qaidam Lake population is clustered together with *A. sinica* (Yin et al. 2011, 2013).

In addition, there are also a number of allegedly bisexual populations, generally based on visual observation and personal communication, in Asian saline habitats, especially in Siberia and China, which have not been identified to any nominal species (Van Stappen et al. 2009; Zheng and Sun 2013). The phylogenetic status of these populations still needs to be determined.

The nomenclatural and taxonomic status of *Artemia parthenogenetica* have been discussed by previous authors (Barigozzi 1980; Belk and Brtek 1995; Baxevanis et al. 2006; see also Table 1). Since Bowen and Sterling (1978), this name has been used in numerous publications (sometimes followed by a population site), whereas some authors preferred to refer parthenogenetic *Artemia* as “populations” (Abatzopoulos et al. 2002a; Baxevanis et al. 2006; Asem et al. 2010). Recent molecular analyses have shown that parthenogenetic *Artemia* is a polyphyletic group, with diploids/triploids being evolutionally close to *A. urmiana* while tetraploids/pentaploids sharing a common ancestor with *A. sinica* (Asem et al. 2016). In another molecular analysis conducted by Eimanifar et al. (2015), the parthenogenetic *Artemia* has been named as “Eurasian Haplotype Complex” comprising a group of putative parthenogenetic *Artemia* lineages which were genetically close to two bisexual species, *A. urmiana* and *A. tibetiana*. The molecular divergence analysis has indicated that a recent population expansion in *A. urmiana* and “Eurasian Haplotype Complex” occurred in the Pleistocene (1.72 Mya) and Holocene (0.84 Mya), respectively. Males of parthenogenetic *Artemia* (produced only by diploids; Saleem Chang et al. 2017) are fertile when mating with bisexual *A. sinica*, *A. tibetiana*, *A. urmiana* and KAZ (Cai 1993; Liu et al. 2007; Maccari et al. 2014), but not fertile when mating with the female of the other bisexual species (MacDonald and Browne 1987). Parthenogenetic females mating with males produce only parthenogenetic offspring (Barigozzi 1974). These results suggest that parthenogenetic *Artemia* may originate from bisexual species native to Asia; the gene pool of bisexual *Artemia* may be affected by diploid parthenogenetic *Artemia* but not vice versa; gene exchange can hardly happen among parthenogenetic individuals of different ploidies. In other words, parthenogenetic individuals cannot readily be considered as belonging to a single species as commented by Abatzopoulos et al. (2002a), and can be assigned to taxa different from bisexuals. There are already some available names referring to parthenogenetic *Artemia*, e.g., *Artemia bivalens* Artom, 1912 (Artom 1912 proposed this name for a tetraploid parthenogenetic population from Capo d’ Istria; also as *Artemia salina bivalens*), and the aforementioned *A. asiatica*, *A. barkolica*, *A. urumuqinica*, *A. ebinurica*, and *A. frameshifta* (?). These names could be candidates to be assigned to parthenogenetic populations, but this requires the type specimens to be re-examined, or/and topotypes to be studied using multidisciplinary methods.

*Artemia* is a taxon of difficult classification because of the lack of discernible characters. Some morphological characters are documented to be useful in delimitate species. For instance, the conical frontal knob on male antenna and the absence of basal spine on gonopod can segregate *A. salina* from the other bisexual species (e.g., Mura and Brecciaroli 2004; Zheng and Sun 2008), the “orthostichous spines” on gonopod are present only in species from China *(A. sinica*, *A. tibetiana*) (Zheng and Sun 2008). Molecular analyses based on genetic markers, like mitochondrial COI and 16SrRNA, and nuclear ITS1 sequences, have successfully resolved the phylogenetic relationships of different populations and are useful to assign a population to a known species/clade (e.g., Baxevanis et al. 2006; Wang et al. 2008; Yin et al. 2011, 2013; Eimanifar et al. 2014, 2015; Asem et al. 2016). However, these methods cannot resolve all taxonomic problems existing in Asian *Artemia*, particularly they cannot tell the taxonomic rank (species/subspecies) of a certain morphological group or phylogenetic clade. As discussed earlier, the test of reproductive isolation, though theoretically supposed to be a gold criterion for diagnosing species, cannot resolve these problems, either. Therefore, based on present knowledge it is hard to tell an exact number of valid species of *Artemia* living in Asia.

As far as we know, there are three ongoing comprehensive projects related to *Artemia* genomics, including the *Artemia* genome project, in which the full nuclear genome of inbred *A. franciscana* from Great Salt Lake was assembled and annotated (Ghent University, Belgium), also a study involving the creation of whole-genome SNP-based second-generation genetic maps (Ocean University of China, China), and a study devoted to complete the mitochondrial genomes of all described bisexual species (including both bisexual clades from Tibet) and parthenogenetic *Artemia* with different ploidy degrees (Hainan Tropical Ocean University, China). We believe that the final results of these studies will further contribute to deciphering the phylogenetic relationships in the genus *Artemia*.
